# Five-Year results of a multi-specialty induction course for surgical training

**DOI:** 10.3389/fsurg.2023.1198696

**Published:** 2023-06-19

**Authors:** Jing Yi Kwan, Panagiotis Lainas, Philippa Banks, Anna Jimenez De Veciana, Hagar Said, Anna Mehrem, Manash Debbarma, Melissa Matthews, Gloria Etim, Chandra Shekhar Biyani, Sanjay Rajpal, Mark Phillipson, Victor Palit, Paul Renwick, Marina Yiasemidou

**Affiliations:** ^1^ Department of Vascular Surgery, Leeds Teaching Hospitals, Leeds, United Kingdom; ^2^ Department of Digestive Surgery, Metropolitan Hospital, HEAL Academic, Athens, Greece; ^3^ Department of Minimally Invasive Digestive Surgery, Antoine-Beclere Hospital, Paris-Saclay University, Clamart, France; ^4^ Department of General Surgery, Bradford Teaching Hospitals, Bradford, United Kingdom; ^5^ School of Medicine, University of York, York, United Kingdom; ^6^ Department of Urology, Mid Yorkshire Hospitals NHS Trust, Wakefield, United Kingdom; ^7^ School of Medicine, Hull York Medical School, Hull, United Kingdom; ^8^ Department of Urology, Leeds Teaching Hospitals, Leeds, United Kingdom; ^9^ Department of Urology, Airedale General Hospital, Airedale, United Kingdom; ^10^ Department of Orthopaedics, Leeds Teaching Hospitals, Leeds, United Kingdom; ^11^ Department of Vascular Surgery, Hull University Teaching Hospitals, Hull, United Kingdom

**Keywords:** teaching, curriculum, surgical training, core surgical training, surgical education

## Abstract

**Background:**

The Essential Surgical Skills Course (ESSC) is a multi-specialty induction “boot camp” style course that has been run successfully for five years. The aim of the current paper is to create an accurate guide for the replication of the course by other teams and assess the course's fitness for purpose, through the survey feedback provided by trainees.

**Methods:**

The course's fitness for purpose was assessed through cumulative five-year survey feedback from trainees. This observational study describes the design and process of content adjustment according to feedback.

**Results:**

The course its five-year span offered twelve different procedural skills in four different specialties. Feedback for each session was persistently >8/10. Key themes identified as beneficial include teacher-to-trainee ratio (often 1:1), teaching style, course structure and responsiveness.

**Conclusions:**

The ESSC was found to be fit for purpose for the induction of trainees into surgical training. The key factors contributing to the success of the course include the structured method of curriculum design, outstanding teaching delivery methods, teacher-to-trainee ratio, the availability of appropriate faculty and infrastructure and the willingness to learn from trainee feedback and adjust the content of the course accordingly. It acts as a paradigm for courses aimed to prepare surgical trainees for a “step-up” in their careers.

## Introduction

Surgical training is one of the longest training programs in the healthcare setting, requiring five to six years of postgraduate training for the full qualification. Within the UK, this is preceded by two years of foundation training and another two years of core surgical training aiming to prepare doctors with the underpinnings of skills required for their specialist interest (1).

There have been long-standing concerns that foundation doctors do not gain significant experience in surgical procedures, resulting in a difficult transition from being a Foundation Year 2 doctor to a Surgical Trainee with associated reduced confidence levels (2). Several factors have contributed to the gap between the Foundation Programme (FP) and Core Surgical Training (CST) becoming more noticeable over the last decades. More junior doctors take time out of training during this time, go into service provision and explore alternative career paths before committing to surgical training (3, 4). There is also a big step up in clinical and procedural skills. The expected surgical skills at the end of FP are minimal, compared to the curriculum of CST (2).

In recent years, it has been reported that both the ability and confidence in procedural skills of surgical doctors at the beginning of their surgical journey have been decreasing (4, 5). Formal teaching is variable across trusts as it is designed and delivered by individual hospitals under supervision from Foundation Schools. Clinical skills teaching is provided mostly on an informal basis (6). As a result, newer cohorts of junior doctors have varying levels of competency in procedural skills once considered “a must-have” (7). This is due to a combination of time constraints (6, 8) and increasing pressures on the NHS tilting the scales more in favour of service provision rather than training and theatre time (9).

The need for formal teaching of surgical and procedural skills has become more intense over the last couple of years. Due to the recent SARS-CoV-2 pandemic and its associated surgical restructuring (10), doctors and surgical trainees globally report restriction of clinical learning opportunities due to the cancellation of cases or “consultant-led operating” policies (11). This posed a significant challenge for surgical educators and drove educators worldwide to develop and embrace new educational methods such as the simulation (11). The use of simulation for training has been explored thoroughly in the last decade, and evidence suggests that skills obtained in the simulation are applicable and transferable to real clinical scenarios (12, 13).

The “Essential Surgical Skills Course (ESSC)/ Bridging the Gap Course (BtG)” was therefore designed to address the above challenges and gaps. The ESSC/ BtG is a multi-specialty surgical, simulation embedded course designed and carried out by Health Education England (HEE) Yorkshire and Humber. The course was inspired by the Association of Surgeons of Great Britain and Ireland (ASGBI) “Management of Surgical Emergencies” a five-day course which had been conducted in sub-Saharan Africa (s-SA) for over three years. Its rationale is to expose future surgical trainees to practice emergency skills and common elective skills encountered in core surgical training.

Embracing the new realities of surgical training, this observational study looks at and describes the development of this course over the span of 5 years. The course has been an educational paradigm, withstanding the test of time, with its educational value growing stronger every year.

## Methods: curriculum design and development

This observational study aims to describe the evolving process of designing and delivering a successful “hands-on” practical course and to assess its fitness for purpose through trainee feedback. Throughout its 5 year span from 2015 to 2019, the course was open to all in-programme training Foundation Year 2 doctors within the Yorkshire and Humber Deanery, United Kingdom, who were interested in pursuing a career in surgery (14). The course was held annually at the Clinical Practice Centre, St James University Hospital, Leeds, West Yorkshire, UK which could be easily reached via public transport from all areas of Yorkshire to ensure accessibility.

The BtG curriculum was developed based on four main considerations. First, a team of experts' clinical experience of what a junior surgical resident will be asked to do during the first year of their core surgical training. Second, key skills listed in the national surgical training curriculum for the core surgical training (ISCP) (15) should be included. Third, foundation year 2 trainees' opinions on what they would like to have been taught prior to and during the course, and finally, reflection on the feedback received from previous years. The intent was to deliver 90% “hands-on” training on models and the rest from didactic lectures.

Each course had 24 participants divided into 4 equal groups which rotated through different surgical skills stations. Each station was staffed with registrars and consultants from the respective specialty with a focus on providing a 1:1 teacher-to-trainee ratio to allow for high-quality, personalized feedback. Each practical skills station was split into two parts. This first part consisted of a demonstration station, which was either a video recording of all taught procedures or a real-life demonstration by a tutor, depending on the availability of resources and varied from year to year. This was followed by a supervised hands-on practice station on simulated models (synthetic or animal tissue). In 2015, the course consisted of solely urology and general surgery skills stations. Orthopaedic stations were added to the curriculum in 2016, and a vascular station (vein patch demonstration and practice) was included from 2018 onwards (Table 1) following feedback.

**Table 1:** Mean scores (out of a maximum of 10) for each skill station from the years 2015 to 2019. Scores were rounded to the nearest 2 decimal places.

Specialty	Skills set (Year included)	2015	2016	2017	2018	2019	*Mean score per station*
Urology	Circumcision	9.38	9.45	9.46	8.50	9.70	9.30
	Suprapubic catheterisation	9.63	9.26	9.47	8.50	9.00	9.17
	Acute scrotum and scrotal fixation	9.29	9.25	9.46	9.00	9.70	9.34
	Scrotal examination	8.00	9.55	9.27	8.5	9.50	8.96
	*Overall mean score for each specialty per year*	9.08	9.38	9.42	8.63	9.48	
General Surgery and Vascular Surgery	Laparoscopic appendicectomy demonstration and practice	9.13	9.57	9.52	9.00	9.15	9.27
	Bowel anastomosis demonstration and practice	9.50	9.63	9.42	9.00	9.75	9.46
	Stoma formation demonstration and practice	9.63	9.42	9.42	8.5	9.30	9.25
	Open hernia repair demonstration and practice	9.00	8.42	N/A	N/A	N/A	8.71
	Vascular vein patch demonstration and practice	N/A	N/A	N/A	8.25	9.45	8.85
	O*verall mean score for each specialty per year*	9.32	9.26	9.45	8.69	9.41	
Trauma and Orthopaedics	Plastering	N/A	9.54	9.47	8.00	8.85	8.97
	Thomas splint	N/A	9.09	8.78	8.00	7.10	8.24
	Plating and wiring of long bones	N/A	9.63	9.52	8.5	8.95	9.15
	*Overall mean score for each specialty per year*	N/A	9.42	9.26	8.17	8.30	

For quality assurance purposes, all trainees were required to complete a mandatory hardcopy feedback form which was handed to each trainee prior leaving the course venue. The survey aimed to collect both quantitative and qualitative feedback. It consisted of a numeric rating scale which requires the trainees to rate the overall educational content of each station on a defined scale out of 10, followed by two open-ended questions—“What went well?” and “What could we have done better?”. In order to obtain meaningful and honest feedback, trainees were not requested to provide any personal details or characteristics to guarantee anonymity.

### Statistics

Mean feedback scores for each station across 5 years was calculated using IBM SPSS Statistics 27.0.

## Results of feedback

### Analysis of quantitative feedback

**Figure 1:**
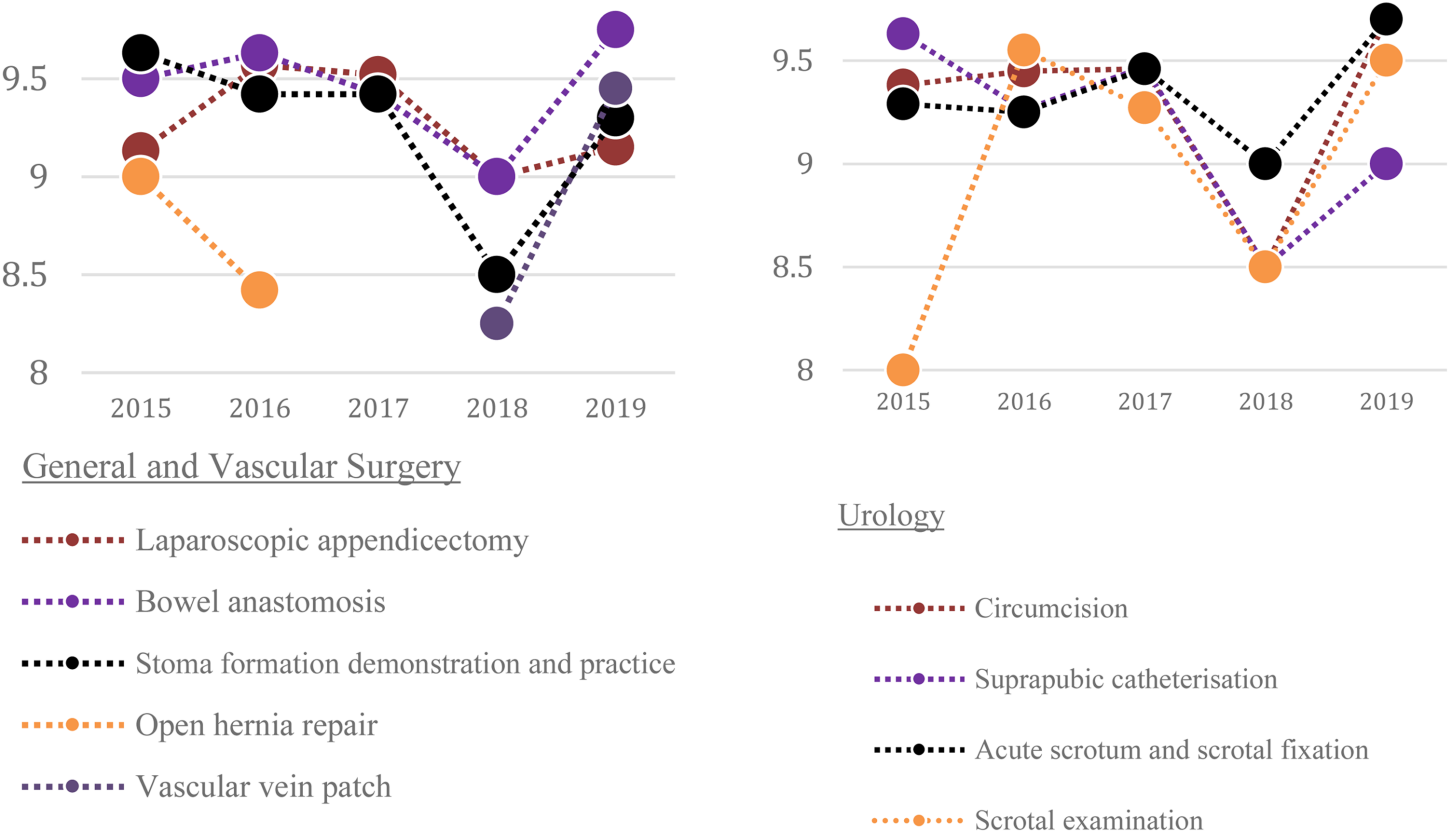
5-year overview for General and Vascular Surgery. **FIGURE 2** 5-year overview for Urology.

**Figure 3:**
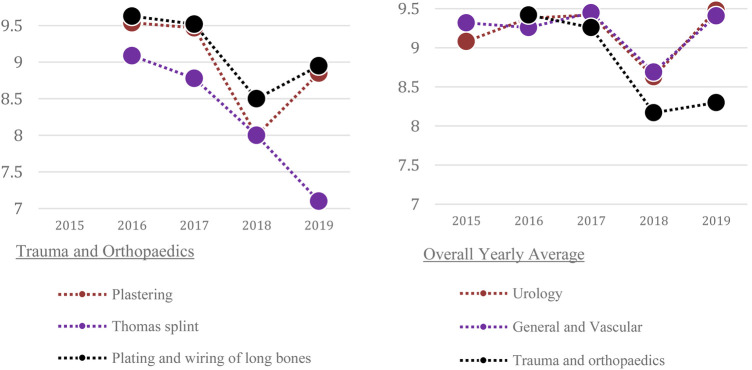
5-year overview for Trauma and Orthopaedics. **FIGURE 4** 5-year overview for Overall Specialty Average.

The development of the teaching curriculum with respective mean feedback scores across 5 years is shown in Table 1 and Figures 1–4. The trend of mean scores demonstrates consistently high scores every year (above 8 out of 10). This suggests that the addition of more specialities and more stations within specialties does not compromise scores and is received favourably amongst participants. Trainee feedback for the different sessions of the course ranged from 8.17–9.42 out of 10 (Table 1).

### Free-text feedback

Free-text comments were predominantly positive. Three areas of positive feedback commonly highlighted by participants were identified. Firstly, participants highlighted that having a spread-out course structure over the two days was ideal as it allowed enough time for each candidate to practise the particular skills—one participant felt that it “was important to keep it over 2 days, as lots of different procedures are taught and then given the opportunity to practise on”, and another felt that it allowed skills to be “taught at your own pace”.

Secondly, most participants appeared to prefer animal tissue compared to plastic models. Within urology stations, the acute scrotum and scrotal fixation station scored the highest mean score of 9.34/10, and it was felt that “it was good to practice on sheep testicles rather than prosthetics.” The general surgery stations were also similarly praised with participants highlighting the “good use of actual bowel to practice on”.

Lastly, many participants highlighted the presence of high-quality tutor ratio as a key component contributing to the course's success. It is important to note that several participants felt that having an informal style of teaching has made their learning experience more enjoyable. Tutors were described as “excellent teachers with good communication skills and non-judgemental feedback”, “enthusiastic and knowledgeable tutors”, and “informal but informative and able to answer any questions”. The informal teaching environment has also allowed participants to “ask questions in a relaxed environment”.

However, some areas of improvement were also raised by the trainees. It was noted that stations that utilized video demonstrations were more poorly received: “The demo video on testicular fixation, everyone in my group struggled to understand the concept of it”, “The demo video on stoma formation was not very clear” and “The video…was very long and had no sound which made it difficult to follow”. This is compared to stations utilising real-life demonstrations which received positive feedback: “…I really enjoyed the live demonstrations…”, and “Good demonstration…”.

## Discussion

The ESSC/BtG course has demonstrated excellent feedback and was well received by trainees. The course was structured according to curriculum requirements but by evolving each year according to trainees' needs and feedback it has managed to grow and consistently deliver highly rated teaching of procedural skills to Foundation Doctors seeking a career in surgical training. For example, it is of note that the open hernia repair demonstration and practice station was discontinued after 2016 after trainee feedback. The latest feedback from 2019 have demonstrated that the current curriculum is well-received, fit for its purpose and has appropriate content for the targeted level of training.

One of the key concepts of the ESSC/BtG course was the emphasis on demonstration as the key teaching style. Within the five classic teaching styles recognised by Grasha in 1994, the “demonstrator” style, also known as the personal model, oversees, guides and directs by showing students how to do things and encouraging students to observe and then emulate the instructor's approach (16). The hands-on nature of the approach which can be observed in all skills stations is long recognised as the main advantage of this teaching style. A disadvantage of this method is that different demonstrators may feel that their approach is the best way, leading to potential conflicting advice. Some students may also struggle with feelings of inadequacy if they feel that they cannot live up to the expected standards that were demonstrated (16). Although it was reassuring that these points were not raised in any of the feedback across 5 years, teachers and trainees should be aware of these potential issues.

Demonstration teaching has historically been used in medicine. The traditional method of teaching in surgery is known as the “See One, Do One, Teach One”, where trainees, after observing a particular procedure once, are expected to be capable of performing that procedure followed by being able to teach another trainee how to conduct that procedure. However, there are concerns that this method should no longer be encouraged due to concerns for patient safety (17). With the increasing use of simulation-based training within the medical and surgical training (18) recent evidence suggests that the current best way to practice in a safe environment is using surgical simulation—demonstration teaching in a low-pressure, stimulating environment without time restraint has been shown to result in higher retention for learners and provides a safe environment to reflect on and learn from mistakes without threat to patient safety or professional identity (17, 18). Based on the success and positive feedback obtained from the course, we encourage organisers of similar surgical-themed courses to consider adopting a similar course format and delivery method.

There are three categories of simulation considered useful for training—(i) inanimate artificial tissues and organs, (ii) fresh tissue or animal models, and (iii) virtual reality and computerised simulation (19). The ESSC/BtG course fully incorporates and utilises the first two- (i) artificial testicular examination models and artificial veins, and (ii) fresh animal tissue including bovine intestines and bull's testicles were used for both general surgery and urology stations. It is of note that the use of anaesthetised animals, particularly pigs, is common in Europe and America with widespread beliefs that practising on animals provides the best training, and progress in medicine is proportional to the availability of good animal models (20). This is in keeping with our findings where participants found the use of animal parts as one of the main merits of the course. Even though the Cruelty to Animals Act of 1876 (21) forbids the use of animals in the UK to gain proficiency in surgical skills, there is no restriction on the use of animal parts in the UK, and pig's trotters are often used to teach suturing and excision biopsy in the Basic Surgical Skills course which is mandatory for all basic surgical trainees and candidates for the MRCS examination. Based on current literature and feedback obtained, we believe that more surgical courses should utilise animal parts for skills teaching where appropriate. On the other hand, the main disadvantages of these models are the cost, and the necessity for purpose-built facilities for cleaning, storage, and disposal (18). Therefore, financial support from the curriculum delivery budgets needs to be ensured to ensure continuity and uniformity of the course yearly. It is also only possible to conduct these sessions in a fully equipped training centre, which could be a significant limiting factor to other educational organisations nationally or internationally.

### Limitations

ECCS/BtG aims to provide an overview of the skills required in surgical specialties that recruit via CST within the UK (general surgery, trauma and orthopaedics, vascular surgery, urology etc). Therefore, a limitation of this course is that it does not include highly-specialised specialties [ear, nose, and throat (ENT), maxillofacial surgery, plastic surgery], or specialties that recruit via a run-through programme (neurosurgery, cardiothoracic surgery, obstetrics and gynaecology). Inclusion of such content is restricted by factors including time, cost, and availability of specific simulators. A pragmatic approach needs to be taken and the final choice of procedure to be included in the final course programme should be tailored according to local and national training requirements.

## Conclusion

The ESSC/ BtG course was well received by Yorkshire and Humber foundation doctors seeking a career in surgery. The results have demonstrated that the current curriculum is fit for its purpose and has appropriate content for the targeted level of training. The key factors contributing to the success of the course include the structured method of curriculum design, outstanding teaching delivery methods, the availability of appropriate faculty and infrastructure and the willingness to learn from trainee feedback and adjust the content of the course accordingly. We encourage surgical training centres within the UK and internationally to consider adopting a similar approach and method of delivery when delivering surgical skills courses.

## Data Availability

The raw data supporting the conclusions of this article will be made available by the authors, without undue reservation.

## Declarations

Kwan is an NIHR Academic Clinical Fellow and Yiasemidou is an NIHR Academic Clinical Lecturer.
